# Ligature of Internal Saphenous Vein

**Published:** 1903-03-14

**Authors:** 


### LIGATURE OF INTERNAL SAPHENOUS VEIN.

This operation is much in favour at present as an accompaniment to the ordinary operation of excision of varicose veins, or, in some hands, as the only operative procedure in certain cases. The operation is such a mild one that it is readily undertaken. Eucaine is quite a sufficient anæsthetic. But a great many of those who have embarked upon the operation must have found themselves somewhat surprised at the difficulty in finding the vein; or, having ligatured what they thought at the time of operation to be the trunk of the internal saphenous vein, the after result must have either made them suspect that only a large tributary has been ligatured or that the operation is in itself useless. The fact is the surgical anatomy of the internal saphenous vein is not sufficiently well known, generally speaking, and the operator has often sought for it in the wrong place. Dr. Campbell[1] draws attention to this, and he has investigated the anatomy of the internal saphenous vein—(1) to discover the possible sources of error which might occur in practising the operation as now taught; (2) to devise, if possible, some guide or landmark which would fix a point at which the vein could be exposed and ligated without possibility of error. The experiments were performed on 50 subjects, and in detail were as follows:—(1) The operation, as now performed, was done on each subject as a preliminary procedure; (2) the skin was reflected back, the vein and its branches exposed, and observations made upon the result of the preliminary ligation; (3) the point at which the vein should be ligated was fixed and its relations to surrounding landmarks observed. The results were interesting. Regarding the preliminary ligature, as usually performed Dr. Campbell succeeded in ligaturing the vein at the proper point in 70 per cent. of the ligations, the other 30 per cent. were distributed between the internal femoral cutaneous, the external femoral cutaneous, the internal saphenous vein below these two branches, and in one instance there were two trunks, one lying above the other, and forming the main trunk about half an inch below the saphenous opening. In 20 per cent. the internal femoral cutaneous was found to have been ligatured. In 6 per cent. of the cases the internal saphenous vein was ligatured below the femoral branches. The external femoral cutaneous was ligatured once, and the anterior portion of the internal cutaneous once when it occurred as a double vein. It was found that the usual incision is too low down and too far towards the inner aspect of the thigh. The average incision was an inch too low and too far in. The proper place to ligature the vein is between its last tributary and the saphenous opening. The length of this portion is about three-quarters of an inch. Dr. Campbell found that the end of a line $3\frac{1}{2}$ inches long, projected from the spine of the os pubis at right angles to Poupart's ligament, always corresponded approximately with the portion of the internal saphenous vein which it was desired to ligate. He recommends an incision parallel with the groin, having its centre at this point.

[1] Medical News, New York, Feb. 21.

